# Interactive Medical Image Segmentation using PDE Control of Active Contours

**DOI:** 10.1109/TMI.2013.2274734

**Published:** 2013-07-24

**Authors:** Peter Karasev, Ivan Kolesov, Karl Fritscher, Patricio Vela, Phillip Mitchell, Allen Tannenbaum

**Keywords:** PDE Control, Reaction-Diffusion, Human-Computer-Interaction, Level Set Methods, Magnetic Resonance Imaging, Computed Tomography, Image Segmentation

## Abstract

Segmentation of injured or unusual anatomic structures in medical imagery is a problem that has continued to elude fully automated solutions. In this paper, the goal of easy-to-use and consistent interactive segmentation is transformed into a control synthesis problem. A nominal level set PDE is assumed to be given; this open-loop system achieves correct segmentation under ideal conditions, but does not agree with a human expert's ideal boundary for real image data. Perturbing the state and dynamics of a level set PDE via the accumulated user input and an observer-like system leads to desirable closed-loop behavior. The input structure is designed such that a user can stabilize the boundary in some desired state without needing to understand any mathematical parameters. Effectiveness of the technique is illustrated with applications to the challenging segmentations of a patellar tendon in MR and a shattered femur in CT.

## I. Introduction

**Figure 1:**
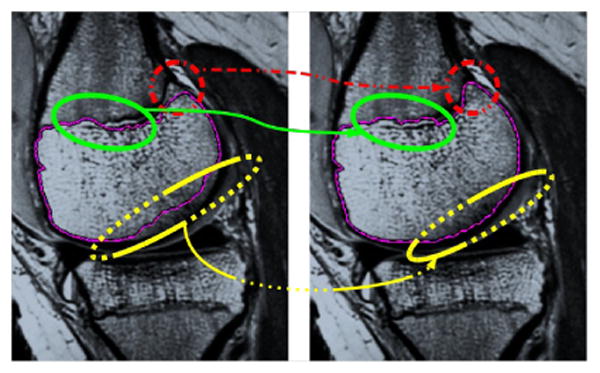
Segmentation by minimizing a meaningful image-dependent functional is not sufficient when the desired anatomic boundary is not actually a minimizer (*left*). An expert user would typically desire to make some corrections (*right*) that contradict the functional's minimizer.

Microwave-frequency Magnetic-Resonance-Imaging (MRI) [1] and X-Ray Computed Tomography (CT) [2] yield three-dimensional volumetric images which are viewed by a medical professional for diagnosis, treatment planning, or population studies [3], [4]. Typically, only a particular anatomic region or organ is of interest and must be *segmented*. Segmentation is the task of identifying and localizing salient structures in the image volume. Since there is an abundance of raw image data that is not analyzed due to the infeasible time and cost of manual segmentation, automated methods for segmentation are the subject of much recent medical computing literature [5]–[7]. However, a human expert's ability to combine observed image data with prior anatomical knowledge to accurately perform segmentation is unmatched by computer algorithms. There is substantial mistrust both from patients and doctors towards fully automatic algorithms. Recognizing this, there has been a recent drive towards interactive segmentation [8]–[10]. Interactive approaches use a data-driven automatic algorithm to process a majority of the volume. As the automatic segmentation runs and displays the current state, a human user can influence the algorithm's behavior to more closely align with an expected result. Fig-1 shows an example of the segmentation corrections a human would make. Ideally, an interactive system should enable a user to create excellent segmentation results with a minimal amount of time and effort.

Interactive medical image segmentation employs software tools such as Seg3D [11] and 3D-Slicer [12] for applying algorithms and visualizing the results; a human expert uses the algorithms to achieve a segmentation that is as close as possible to the ideal region boundary. Available algorithms include iterative methods with a concept of *time*; a partial differential equation (PDE) is used to model the space-time relationship between image data and segmentation boundaries [13]–[16]. Given user-specified parameters and initial state, incremental modifications are made to the segmentation and shown to the user [17]–[20]. It is not clear to a user whether it is possible for their ideal region boundary to be a steady-state. In practice, they must stop the algorithm at some time *t_f_* when the segmentation is reasonably accurate, then apply smoothing and manual corrections to the boundary. An alternative approach is to formulate segmentation as a time-independent problem; in [21]–[23], user input acts as a constraint in finite-dimensional nonlinear optimization problems. It is not known a-priori whether the user's ideal region boundary is a feasible solution for some collection of constraints, while a changing number of user input constraints can affect the computational complexity. Furthermore, the time-independent formulation can lead to large changes in the segmentation output when new user input is received. Other classes of algorithms such as graph-cuts [19], [24], [25] are also effective for automated segmentation; we consider only level-set PDE algorithms due to their theoretical compatibility with methods in the PDE control literature.

Level-set methods define a region boundary implicitly as the zero level-set of a function *ϕ*(**x**, *t*) with domain^1^ Ω ⊂ ℝ*^n^*. Temporal changes of the region boundary are described by a partial differential equation (PDE) as a consequence of the implicit representation. Typically $\frac{\partial \phi }{\partial t}$ arises as the gradient flow that minimizes some meaningful functional of *ϕ* and the image data *I*(**x**). From a controls perspective, the image-dependent PDE is an open-loop system. We present a framework for interactive segmentation using feedback augmentation of a level set PDE system; the results and theory are a substantial extension of the preliminary version [26]. This paper is motivated by the following observation: when human users influence the level-set evolution, *they have in mind a desired reference state and are trying to apply control to an image-dependent PDE system*.

In the literature on control of PDE systems, two characteristics of problems are the domain of input actuation and the available measurement (pointwise throughout a domain, boundary-value only, or as an integral over space). Control through region boundaries is of paramount interest; [27] characterizes the stabilizing controls and admissible boundary conditions for a class of unstable reaction-diffusion (R-D) systems. Similarly, [28] explicitly computes invariant regions for coupled R-D systems. Stabilization of the viscous Burgers equation $u_{t}+\frac{d}{\text{dx}}(\frac{1}{2}u^{2})=\varepsilon u_{\text{xx}}$ using boundary actuation is achieved in [29], [30] by first designing a feedback law using *u*(**x**, *t*) over all **x**, then deriving a *u*-observer that uses only boundary measurements. In [31], the inviscid $u_{t}+\frac{d}{\text{dx}}(\frac{1}{2}u^{2})=0$ is stabilized with a boundary input *u*(0, *t*) from an admissible set of controls that admits a weak solution to the initial-boundary-value problem. A common theme in PDE-control for setting input and gain values are scalar functions *w*(*t*) defined as functional of the state *u*(*x*, *t*) [32]–[34]; *e.g.*, *w*(*t*) = ∫_Ω_ *k*(*x*, *t*)*u*(*x*, *t*)*dx*. Such inputs appear in this paper as well, with image dependence entering via a term analogous to *k*(*x*, *t*). The model used in this paper uses actuation and measurement within a neighborhood of the time-varying segmentation boundary.

### Contribution

Using a PDE formulation guarantees that the computational complexity is fixed and that the segmentation result changes continuously over time. By incorporating control-theoretic tools, it is shown that the steady state segmentation can be driven to an ideal reference boundary. Input from the human user indicating locations where the current state does not match the desired reference state is processed and used as feedback in the PDE. An approach similar to backstepping [33], [35] is used; first, stability of a labeling error functional is shown under the assumption of a known reference state. Second, an auxiliary observer-like system that reacts to user input is formulated. The net coupled system is shown to have bounded error when a sufficient amount of user input has been accumulated. To the best of our knowledge, this is the first approach to interactive level set segmentation with input from the user used in feedback to guarantee stabilization about a reference boundary.

### Organization

The remainder of this paper is as follows.

**Figure 2:**
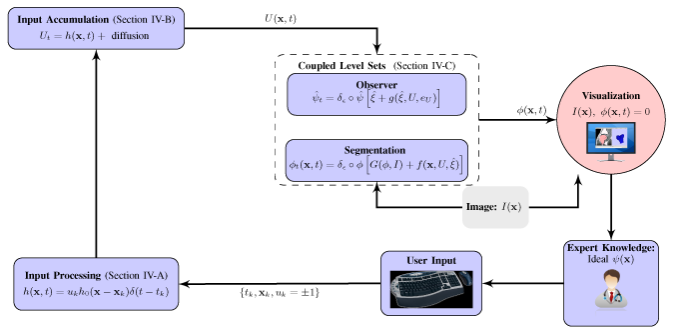
Block diagram of the proposed control formulation. Feedback compensates for deficiencies in automatic segmentation by exploiting the human expert's interpretation of complex imagery.

Image segmentation based on the narrow-band level-set method is reviewed in Section II. Following an approach similar to back-stepping, a controller is proposed in Section III that stabilizes a labeling-error term, assuming exact knowledge of a reference state. An auxiliary observer-like system is designed in Section IV to process a user input signal and estimate the reference state. Application of the technique to interactive segmentation of CT and MRI volumes is demonstrated in Section V, using images that are difficult to segment with existing methods. The proposed algorithm is compared to related methods in Section VI. Section VII summarizes the results and applicability of the approach. Key components of the final closed-loop system and corresponding paper sections are visualized in Fig-2.

## II. Level Sets and Automated Segmentation

The class of open-loop systems considered in this work are PDE-based segmentation algorithms using the popular *level set method*, reviewed in Section II-A. Section II-B describes the limitations of open-loop segmentation, thus motivating the feedback control model of interactive image segmentation in Section III-A.

### A. Review of Level Set Methods

Level set methods represent time-varying region boundaries in a computationally straight-forward manner [36], [37]. Define Ω ⊂ ℝ*^n^* to be the spatial domain and x a coordinate in Ω. Labeling assignments are represented with an implicit function *ϕ*(**x**, *t*) : Ω x [0, *t*) → ℝ. Boundaries between regions of interest are represented as level sets where *ϕ*(**x**, *t*) = *C*. Propagation of *ϕ* over time is defined by *ϕ_t_* = − 〈∇*ϕ*, **f**〉 for some vector field **f** that is a function of image data, of *ϕ*, and spatial derivatives of *ϕ*. In this paper, *ϕ*(**x**, *t*) > 0 denotes the interior of a segmented region and the (outward) normal vector N along a level set of *ϕ* is $N=-\frac{\nabla \phi }{\left|\nabla \phi \right|}$. Assuming the gradient and normal of *ϕ* exist, the general form of a level set PDE is

(1) $$\phi_{t}=\;|\nabla \phi |\langle N,f\rangle ≐F|\nabla \phi |.$$

In “variational level set methods” [38]–[40], the *F* in Eq-1 is constructed to regulate some quantity of the form

(2) $$E(t)=\int_{\Omega }g(x,\phi ,\nabla \phi )dx.$$

As pointed out in [41], many image segmentation applications use an *artificial* time parameter *t*, which arises solely due to an iterative minimization of Eq- 2. In this paper, however, *t* corresponds to a physical time since the human user watches a time-varying visualization of *ϕ*(**x**, *t*) to decide where and when to provide input.

It is usually desirable to have |*ϕ_t_*| > 0 only in a neighborhood of the moving zero level set [42]–[44]. This *narrowband* restriction is used in the image processing community for several reasons. First, efficient numerical techniques such as the “sparse field method” [45] update *ϕ* only within the narrow-band region. Dimensions of 3D medical image volumes are on the order of 512^3^; real-time performance on desktop computers is attainable only by restricting computations to a subset of Ω. Second, algorithms typically seek to separate Ω into statistically different regions within the *I*(**x**) image. It is sufficient to know only sign(*ϕ*) when labeling regions as interior and exterior; the magnitude of *ϕ* has little meaning in segmentation applications. Finally, if *ϕ_t_* is nonzero for arbitrary values of |*ϕ*|, zero crossings can develop far from the initial *ϕ* = 0 level-set; a visualization of *ϕ* would show new “boundaries” spontaneously appearing. Users will be confused by such behavior; they expect to initialize *ϕ*(**x**, 0) and watch a moving level set.

**Figure 3:**
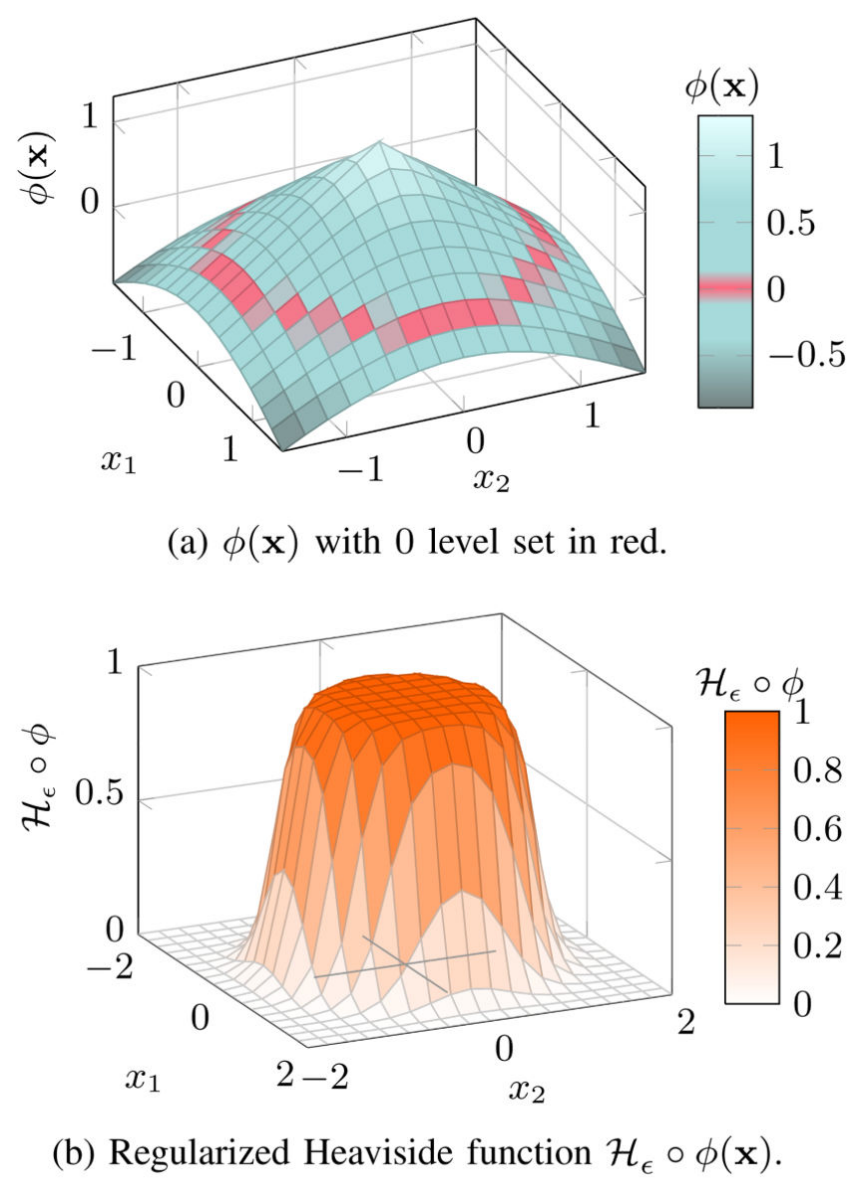
The interior of a segmented region satisfies *ϕ*(**x**) > 0 while its exterior has *ϕ*(**x**) < 0.

Ω is divided into *exterior* and *interior* regions by a regularized version of the Heaviside step function denoted by 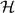
*_ε_*, illustrated in Fig-3. This regularized step function and its derivative *δ_ε_* are defined as

(3) $$H_{\varepsilon }\circ \phi =\left\{ \begin{array}{ll} 1 & \text{if}\;\phi >\varepsilon \\ 0 & \text{if}\;\phi <-\varepsilon \\ \frac{1}{2}(1+\frac{\phi }{\varepsilon }+\frac{1}{\pi }\sin(\frac{\pi \phi }{\varepsilon })) & \text{otherwise} \end{array}\right.$$

(4) $$\delta_{\varepsilon }\circ \phi =\left\{ \begin{array}{ll} 1/\varepsilon & \text{if}\;\phi =0\\ 0 & \text{if}\;|\phi |\;>\varepsilon \\ \frac{1}{2\varepsilon }(1+\cos(\frac{\pi \phi }{\varepsilon })) & \text{otherwise} \end{array}\right.$$

This paper considers systems described by narrowband level-set PDEs with the general form

(5) $$\phi_{t}=\delta_{\varepsilon }\circ \phi \left[G(H_{\varepsilon }\circ \phi ,I)+\lambda \nabla \cdot \frac{\nabla \phi }{\left|\nabla \phi \right|}\right],\phi (x,0)=\phi_{0}(x).$$

Application-specific goals, such as minimization of a functional, dictate the choice of image-dependent *G*(·) in Eq-5; an example is given in the next section. A concise notation for the elliptic operator $\nabla \cdot \frac{\nabla F}{\left|\nabla F\right|}$ is *κ*(*F*), where *F* is a smooth function with non-vanishing gradient defined on **x** ∈ Ω.

In practice, the nonlinear PDEs encountered in level-set segmentation will tend to develop discontinuities. Periodic reinitialization of *ϕ* to a signed distance function [37], [46] mitigates these effects while preserving the *ϕ* = 0 level set. Re-distancing enforces the properties

(6) $$0<p_{1}<\left|\nabla \phi \right|<p_{2},-\kappa_{0}<\kappa (\phi )<\kappa_{0},$$

where *p*_1_, *p*_2_, and *κ*_0_ are fixed constants for a given re-distancing method. These bounds are helpful for control synthesis in Section III.

### B. Segmentation as an Open-Loop System

Automated image segmentation systems are designed under the assumption that a particular discriminative model captures distinguishing features that separate regions of interest from the rest of the image volume. Consequently, the systems often lead to erroneous segmentation results when their underlying assumptions do not hold. The term “open-loop” in the context of automatic segmentation means that the system evolves without any external input that might indicate failure of model assumptions and that the boundary is not moving towards a desired steady-state. Such systems arise from discriminative statistical models in the literature, wherein functionals are proposed that either maximize statistical differences between an object and its background or maximize similarity of the object to a template [40]. Several examples of statistical quantities used are region-based feature means [14], feature covariance [47], and *n*-dimensional non-parametric density estimates [48], [49].

Nevertheless, a recurring problem is that many objects of interest do not coincide with minima of a proposed functional. As an example, consider segmentation via the “mean-alignment” system [14]. The weighted means of image *I*(**x**) in the interior (*ϕ* > 0) and exterior (*ϕ* < 0) regions are

(7) $$\mu_{\text{in}}≐\frac{\int_{\Omega }\left[H_{\varepsilon }\circ \phi \right]I\;dx}{\int_{\Omega }[H_{\varepsilon }\circ \phi ]dx},\;\mu_{\text{out}}≐\frac{\int_{\Omega }\left[1-H_{\varepsilon }\circ \phi \right]I\;dx}{\int_{\Omega }\left[1-H_{\varepsilon }\circ \phi \right]dx}.$$

A gradient flow for the functional

(8) $$E(t)=\frac{1}{2}\int_{\Omega }H_{\varepsilon }\circ \phi {\left(I-\mu_{\text{in}}\right)}^{2}+(1-H_{\varepsilon }\circ \phi ){\left(I-\mu_{\text{out}}\right)}^{2}dx+\lambda \int_{\Omega }\left|\nabla H_{\varepsilon }\circ \phi \right|dx$$

gives the following narrowband open-loop system:

(9) $$\begin{array}{ll} \phi_{t} & =\delta_{\varepsilon }\circ \phi [-{\left(I-\mu_{\text{in}}\right)}^{2}+{\left(I-\mu_{\text{out}}\right)}^{2}+\lambda \kappa (\phi )],\\ \phi (x,0) & =\phi_{0}(x). \end{array}$$

**Figure 4:**
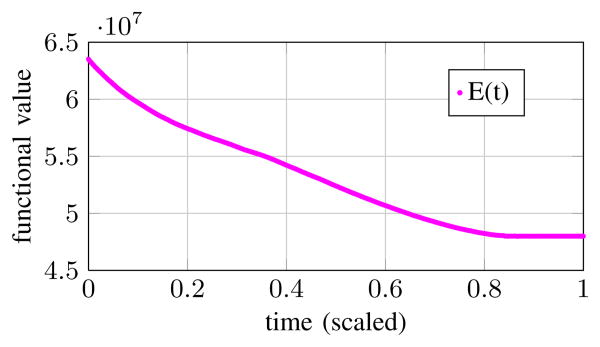
*E*(*t*) functional (Eq- 8) regulated by the open-loop system. Corresponding images shown in Fig-5.

**Figure 5:**
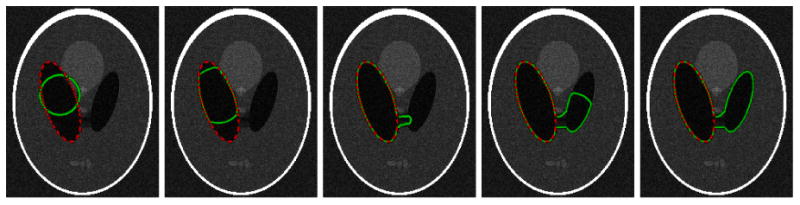
Desired segmentation and {*ϕ*(**x**, t) = 0} level-set overlayed on image *I*(**x**). A user would like to segment the “left ventricle” in this synthetic image that resembles an MRI scan of the brain. Evolving *ϕ* according to Eq-9 shrinks *E*(*t*) successfully (Fig4). However, the open-loop system fails to segment the desired region.

Fig4 and Fig-5 illustrate the behavior of open-loop system Eq-9 on a synthetic image that resembles a noisy image of the left and right ventricles in the brain. The desired segmentation boundary is drawn in dashed red, while the moving zero level set is solid green. Although the open-loop system correctly stops along most of the reference boundary, it creeps into an adjacent region of *I*(**x**) in the course of minimizing *E*(*t*).

## III. Feedback Augmentation of a Narrowband Levelset PDE

### A. Reference State and Input Structure

Rather than having the human user give up on the PDE system and manually outline the desired region of interest, the PDE can be augmented with a user-driven control input. A control solution is sought due to limitations in the efficacy of open-loop system Eq- 5 for real images. Necessary human effort in segmentation can then be kept low; the user need not apply input in locations where the open-loop system keeps *ϕ* in agreement with a desired segmentation.

Let *ψ* denote the ideal reference segmentation. A human user could manually trace the level set *ψ*(**x**) = 0 if given unlimited time. We seek to drive *ϕ* towards an explicit estimate of *ψ*, while maintaining closed-loop stability and minimizing burden placed on the user. User-driven control effort should preserve the advantages of the narrowband formulation noted in Section II-A; therefore, an admissible control signal *f*(**x**, *t*) will act only on the |*ϕ*| ≤ *ε* subdomain of Ω. The closed-loop system then becomes

(10) $$\phi_{t}=\delta_{\varepsilon }\circ \phi [G(\phi ,I)+\lambda \kappa (\phi )+f(x,t)].$$

In this section, *ψ*(**x**) is assumed known; a control is synthesized to drive *ϕ* such that it matches *ψ*. Later in Section IV, a coupled system that estimates *ψ* from available user input is formulated.

### B. Existence of a Regulatory Control

Define the pointwise and total labeling error as *ξ* and 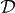, respectively:

(11) $$\xi (x,t)≐(H_{\varepsilon }\circ \phi -H_{\varepsilon }\circ \psi ),\;D(t)≐\frac{1}{2}\int_{\Omega }\xi^{2}dx.$$

If *ψ* is known and available, regulation of 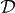
(*t*) is straightforward with the following two theorems in this section. The regulatory control uses known bounds on the image-dependent *G* term of Eq-10; define *G*_M_(**x**), *G̅*_M_ to be upper bounds such that for any segmentation state *ϕ*,

(12) $$|G(\phi (x),I(x))|\leq G_{M}(x)\leq {\bar{G}}_{M}.$$

**Theorem III.1** Using a spatially-varying *U*(**x**), a control for Eq-10 that stabilizes functional Eq-11 is

(13) $$f(x,t)=-\xi U^{2}+\lambda (t)\;[\kappa (\delta_{\varepsilon }^{2}\circ \phi \cdot \xi )-\kappa (\phi )].$$

Given constants λ_0_ > 0, λ_1_ > 0, *ρ* ∈ (0, 1), a sufficient condition for boundedness of 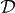 is

(14) $$\lambda (t)=\lambda_{0}+\lambda_{1}D,\text{and}\left|U(x)\right|\geq {\left(\frac{G_{M}(x)}{\rho }+1\right)}^{1/2}≐U_{M}.$$

Furthermore, when the error *ξ* is large in the sense of

(15) $$\rho \int_{\Omega }\delta_{\varepsilon }^{2}\circ \phi \left|\xi \right|\text{dx}\leq \int_{\Omega }\delta_{\varepsilon }^{2}\circ \phi \;\xi^{2}\mathrm{dx},$$

the rate of convergence is bounded by

(16) $$\frac{d}{\text{dt}}D\leq -\int_{\Omega }\delta_{\varepsilon }^{2}\circ \phi \;\xi^{2}dx\leq 0.$$

*Proof:* Re-arranging terms in 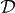
′ and integrating by parts making use of *δ_ε_* ∘ *ϕ* = 0 on ∂Ω:

(17) $$\frac{d}{\text{dt}}D=\int_{\Omega }\xi \dot{\xi }\text{dx}=\int_{\Omega }\xi \delta_{\varepsilon }\circ \phi \cdot \phi_{t}\text{dx}$$

(18) $$=\int_{\Omega }\delta_{\varepsilon }^{2}\circ \phi [\xi G(I,\phi )-{\left(\xi U\right)}^{2}]\text{dx}+\lambda \int_{\Omega }\delta_{\varepsilon }^{2}\circ \phi \xi \nabla \cdot \frac{\nabla \delta_{\varepsilon }^{2}\circ \phi \xi }{\left|\nabla \delta_{\varepsilon }^{2}\circ \phi \xi \right|}\text{dx}$$

(19) $$=\int_{\Omega }\delta_{\varepsilon }^{2}\circ \phi [\xi G(I,\phi )-{\left(\xi U\right)}^{2}]\text{dx}-\lambda \int_{\Omega }\nabla (\delta_{\varepsilon }^{2}\circ \phi \xi )\cdot \frac{\nabla (\delta_{\varepsilon }^{2}\circ \phi \xi )}{\left|\nabla (\delta_{\varepsilon }^{2}\circ \phi \xi )\right|}\text{dx}$$

(20) $$=\int_{\Omega }\delta_{\varepsilon }^{2}\circ \phi \xi G(I,\phi )\text{dx}-\int_{\Omega }\delta_{\varepsilon }^{2}\circ \phi {\left(\xi U\right)}^{2}\text{dx}-\lambda \int_{\Omega }|\nabla (\delta_{\varepsilon }^{2}\circ \phi \xi )|\text{dx}.$$

The Poincaré inequality in *L*^1^ guarantees the existence of a constant *r* such that

(21) $$\int_{\Omega }\left|\delta_{\varepsilon }^{2}\circ \phi \;\xi \right|\text{dx}\leq r\int_{\Omega }|\nabla (\delta_{\varepsilon }^{2}\circ \phi \;\xi )|\text{dx},$$

(22) $$-\int_{\Omega }|\nabla (\delta_{\varepsilon }^{2}\circ \phi \;\xi )|\text{dx}\leq -\frac{1}{r}\int_{\Omega }\delta_{\varepsilon }^{2}\circ \phi \left|\xi \right|\text{dx},$$

where *r* is at most half the diameter of Ω [50]. Substituting Eq- 22 into Eq- 19 bounds the Lyapunov functional's time derivative:

(23) $$\frac{d}{\text{dt}}D\leq \int_{\Omega }\delta_{\varepsilon }^{2}\circ \phi [\xi G(I,\phi )-{\left(\xi U\right)}^{2}]\text{dx}-\frac{\lambda }{r}\int_{\Omega }\delta_{\varepsilon }^{2}\circ \phi \left|\xi \right|\text{dx}.$$

The case of |*ξ*| being large relative to *ρ* in an integral sense (Eq-15) also implies

(24) $$\int_{\Omega }\delta_{\varepsilon }^{2}\circ \phi |\xi G|\text{dx}\leq \int_{\Omega }\delta_{\varepsilon }^{2}\circ \phi \left|\xi \right|\frac{\left|\xi \right|}{\rho }G_{M}\text{dx}.$$

Substituting the condition on |*U*(**x**)| magnitude from Theorem III.1 gives

(25) $$\begin{array}{r} \int_{\Omega }\delta_{\varepsilon }^{2}\circ \phi \left|\xi \right|\frac{\left|\xi \right|}{\rho }G_{M}\text{dx}<\int_{\Omega }\delta_{\varepsilon }^{2}\circ \phi \xi^{2}\left(\frac{G_{M}}{\rho }+1\right)\text{dx},\\ \int_{\Omega }\delta_{\varepsilon }^{2}\circ \phi \xi^{2}\left(\frac{G_{M}}{\rho }+1\right)\text{dx}\leq \int_{\Omega }\delta_{\varepsilon }^{2}\circ \phi {\left(\xi U\right)}^{2}\text{dx}, \end{array}$$

and the error rate 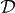
′ is negative semidefinite with a bound

(26) $$\frac{d}{\text{dt}}D\leq -\int_{\Omega }\delta_{\varepsilon }^{2}\circ \phi \xi^{2}\text{dx}\leq 0.$$

Boundedness of 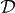 is established after substituting the λ proposed in Theorem III.1:

(27) $$D^{\prime }\leq \int_{\Omega }\delta_{\varepsilon }^{2}\circ \phi \xi \text{Gdx}-\frac{\lambda }{r}\int_{\Omega }\delta_{\varepsilon }^{2}\circ \phi \left|\xi \right|\text{dx}$$

(28) $$\leq {\bar{G}}_{M}\int_{\Omega }\delta_{\varepsilon }^{2}\circ \phi \left|\xi \right|\text{dx}-\frac{1}{r}(\lambda_{0}+\lambda_{1}D)\int_{\Omega }\delta_{\varepsilon }^{2}\circ \phi \left|\xi \right|\text{dx}$$

(29) $$\leq ({\bar{G}}_{M}-(\lambda_{0}+\lambda_{1}D)/r)\int_{\Omega }\delta_{\varepsilon }^{2}\circ \phi \left|\xi \right|\text{dx}.$$

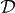
′ > 0 is only possible for 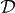 < (*rG̅*_M_ − λ_0_) /λ_1_, with 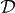
′ ≤ 0 otherwise. Thus, 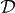 is bounded.

**Remark** A near-optimal (i.e. low) value for *r* can be obtained by substituting the definitions of δ*_ε_*, 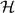
*_ε_* (eqns. 4,3) into $\left|\nabla (\delta_{\varepsilon }^{2}\circ \phi \;\xi )\right|$, applying the chain rule, and comparing to $\left|\delta_{\varepsilon }^{2}\circ \phi \;\xi \right|$ (omitted for space). In practice, *r* can be directly estimated via numerical evaluation of the integrals in Eq- 21 and is on the order of the |∇*ϕ*| due to re-distancing.

## IV. Auxiliary System Design

In the previous section, a stabilizing controller was developed that drives the segmentation towards a reference state relying on fixed quantities *ψ* and *U*, which are not known in practice. The user will be employed to provide the missing information by occasionally applying discrete corrections to the segmentation; these corrections are accumulated over time. From the current segmentation and user input, an estimate of the ideal *ψ* must be inferred. These considerations lead to the coupled dynamical system presented in this section. A method for processing discrete input from a mouse or stylus to generate a distributed *U* is proposed in Section IV-A, while the accumulation of input is addressed in Section IV-B. An observer-like system is formulated in Section IV-C to compute *ψ̂*, the explicit estimate of ideal state *ψ*.

### A. User Input Processing

**Figure 6:**
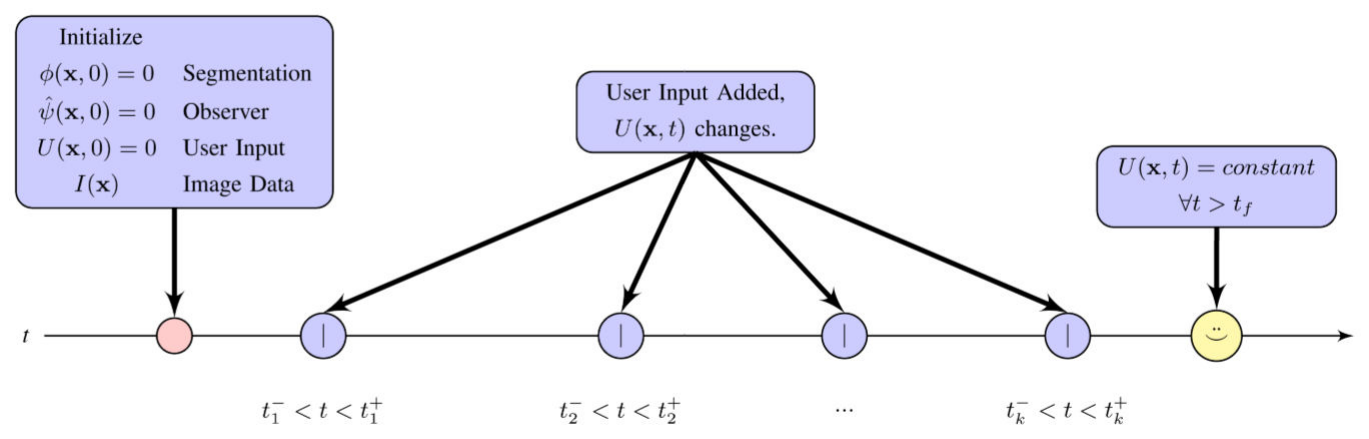
After initialization, the inner loop of Fig-2 updates *ϕ* and *ψ*. Input from a human user applies impulses at times *t_k_* that accumulate as *U*(**x**, *t*). Between times *t_k_*, the inner loop changes steady-state in response to updated *U*(**x**, t). The user stops applying input when the visualization of *ϕ*(**x**, *t*) is satisfactory.

Raw input from the user arrives in the form of binary decisions as to whether a given location in space is correctly labeled as inside or outside the segmentation boundary. The user clicks with a mouse or stylus at discrete points in Ω and time, as illustrated in Fig-6. Define *t_k_*, *k* ∈ ℕ to be the sequence of times at which the user sees a visualization of *ϕ* and has an opportunity to apply input. At time *t_k_*, they look at the labeling of *ϕ*(**x***_k_*, *t_k_*) and either *(a)* apply a signed impulse denoting a “vote” for setting the label there or *(b)* do nothing because they agree with the current labeling of *ϕ*(**x***_k_*, *t_k_*). Denote these sequential actions as

(30) $$u_{k}=\left\{ \begin{array}{cc} +1 & \text{if}\;\psi (x_{k})>0>\phi (x_{k},t_{k}),\\ -1 & \text{if}\;\psi (x_{k})<0<\phi (x_{k},t_{k}),\\ 0 & \text{otherwise}. \end{array}\right.$$

Before these inputs can be accumulated into *U*, they must be mapped into the space-time domain with some fixed support Define the function *h*(**x**, t) as

(31) $$h(x,t)≐\sum_{k}u_{k}h_{0}(x-x_{k})\delta (t-t_{k}),$$

where *h*_0_(·) is a weight function and *δ*(*t* − *t_k_*) is the Dirac delta As noted in [21] using an image-dependent metric for *h*_0_(·) is a useful way to weight spatial distances The examples in this paper use

(32) $$h_{0}(\cdot )≐\left(\frac{\sigma_{I}^{2}}{\sigma_{I}^{2}+|I(x)-I(x_{k})|}\right)\exp\left(\frac{-{\left\|x-x_{k}\right\|}_{2}^{2}}{\sigma_{x}^{2}}\right),$$

which incorporates both Euclidean distance from **x** to **x***_k_* and similarity between image values at *I*(**x**) and *I*(**x***_k_*).

### B. Accumulation of User Input

The label error impulse inputs accumulate over time to define the control *U*. However *U* must be regulated to prevent excessive input magnitudes while ensuring spatial smoothness and enabling |*U*| ≥ *U*_M_ to satisfy the conditions of Section III-B An undesirable excess *U* and |∇*U*| can occur in *U_t_* = *h*(**x**, *t*) because the human user causes *h*(**x**, *t*) without understanding how their “vote” inputs influence the segmentation dynamics Furthermore when the label-error is shrinking at a consistent rate but over a large area it is expected that the human user will be impatient and apply excess input magnitude in an attempt to speed up the moving *ϕ* = 0 boundary.

Regulation of *U* is achieved using a nonlinear diffusion process together with accumulation of *h*(**x**, *t*):

(33) $$\frac{\partial U}{\partial t}=h(x,t)+\nabla \cdot [H_{\varepsilon }\circ ({\left(U/U_{M}\right)}^{2}-1)\nabla U],U(x,0)=0.$$

Changes in *U* are dominated by *h*(**x**, *t*) for |*U*| ≪ *U*_M_. As |*U*| grows, the diffusion coefficient 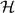
*_ε_* ∘ ((*U*/*U*_M_)^2^−1) gains influence. The following example illustrates qualitative behavior of the *U* system.

**Figure 7:**
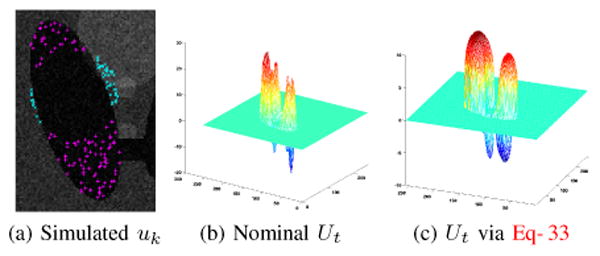
Regulating the input-integration with nonlinear diffusion keeps *U* smooth and bounded. Diffusion occurs when inputs *u_k_* occur in excess of *U*_M_; here, *U*_M_ = 10.

**Example** Consider the two-dimensional image slice shown in Fig-7a. A simulated “user” chooses locations **x***_k_* at which an update *h*(**x**, *t*) is applied according to Eq-31. Blue ‘×’ and red ‘+’ denote places where *h*(**x**, *t*) is negative and positive, respectively. Fig-7b shows what *U* would look like without nonlinear diffusion. Fig-7c shows the response of the regulated *U*-system from Eq- 33. Comparing 7b and 7c, it is clear that the latter is smoother and satisfies *U* ≤ *U*_M_.

### C. Label-Error Estimate Dynamics

Dynamically estimating the reference state necessitates a coupled system; *ϕ* and the estimate of *ψ* evolve simultaneously Let *ψ̂*(**x**, *t*) be an estimate for *ψ*(**x**) and define the error terms

(34) $$\hat{\xi }≐H_{\varepsilon }\circ \phi -H_{\varepsilon }\circ \hat{\psi },\;e_{U}≐H_{\varepsilon }\circ \hat{\psi }-H_{\varepsilon }\circ U.$$

Feedback in the *ϕ* system (Eq-10) will now use *ξ̂*,

(35) $$\begin{array}{cc} \phi_{t} & =\delta_{\varepsilon }\circ \phi \left[G(\phi ,I)+\lambda \kappa (\phi )+f(x,U,\hat{\xi })\right]\\ \phi (x,0) & =\phi_{0}, \end{array}$$

where the initial *ϕ*_0_ is specified by the user The auxiliary *ψ̂*(**x**, *t*) observer-like system is driven by accumulated user input *U* together with error terms *ξ̂* and *e_U_*:

(36) $$\begin{array}{cc} {\hat{\psi }}_{t} & =\delta_{\varepsilon }\circ \hat{\psi }\left[\hat{\xi }+g(\hat{\xi },U,e_{U})\right]\\ \hat{\psi }(x,0) & =\phi (x,0). \end{array}$$

Total labeling error is defined by the following functionals of *ξ̂* and *e_U_*, where *α* is a constant parameter:

(37) $$\text{observer vs. user input}:F(t)≐\int_{\Omega }\frac{1}{2}{\left(\alpha U\right)}^{2}e_{U}^{2}dx,$$

(38) $$\text{observer vs. visualized state}:\hat{D}(t)≐\int_{\Omega }\frac{1}{2}{\hat{\xi }}^{2}dx.$$

In addition to stabilizing 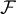 + 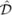, the control proposed in Theorem IV.1 is designed to achieve a useful qualitative behavior. When the user is satisfied with the agreement between *ϕ*(**x**, *t*) and their ideal *ψ*(**x**), it is assumed that *U*(**x**) remains constant; either the user never needed to apply a correction near **x** or has otherwise stopped adding more inputs. In this case, *ψ̂* should follow *ϕ*. Conversely, when *U*(**x**) grows due to persistent human input, *ψ̂* is to become increasingly driven towards *U* irrespective of agreement between *ψ̂* and *ϕ*. Subsequently, *ψ̂* should pull *ϕ* along due to the coupling term −*ξ̂U*^2^ (Eq. 13) in the closed-loop dynamics of *ϕ* .

**Theorem IV.1** Let *g*(*ξ̂*, *U*, *e_U_*) = −*e_U_*(*α*U)^2^ and consequently

(39) $${\hat{\psi }}_{t}=\delta_{\varepsilon }\circ \hat{\psi }\left[\hat{\xi }-e_{U}{\left(\alpha U\right)}^{2}\right].$$

Assume that user input has stopped (*U* remains constant) and Theorem III.1 is satisfied. Then, the sum *V*(*t*) ≐ 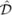 + 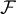 has a negative semidefinite time derivative:

(40) $$V^{\prime }(t)\leq -{\int_{\Omega }\delta_{\varepsilon }^{2}\circ \phi \;{\hat{\xi }}^{2}\text{dx}-\int_{\Omega }\delta_{\varepsilon }^{2}\circ \hat{\psi }\left[{\left(\alpha U\right)}^{2}e_{U}-\hat{\xi }\right]}^{2}\text{dx}.$$

Proof:

Computing the time derivative *V*′(*t*) = 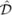
′> + 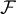
′,

(41) $$F^{\prime }=\int_{\Omega }{\left(\alpha U\right)}^{2}e_{U}{\dot{e}}_{U}dx=\int_{\Omega }{\left(\alpha U\right)}^{2}e_{U}(\delta_{\varepsilon }\circ \hat{\psi }\cdot {\hat{\psi }}_{t})dx$$

(42) $$\hat{D^{\prime }}=\int_{\Omega }\hat{\xi }\dot{\hat{\xi }}dx=\int_{\Omega }\hat{\xi }(\delta_{\varepsilon }\circ \phi \cdot \phi_{t}-\delta_{\varepsilon }\circ \hat{\psi }\cdot {\hat{\psi }}_{t})dx.$$

Substituting for *ϕ_t_* and *ψ̂_t_*,

(43) $$F^{\prime }=-\int_{\Omega }\delta_{\varepsilon }^{2}\circ \hat{\psi }{\left(\alpha U\right)}^{2}\left[e_{U}^{2}\alpha^{2}U^{2}-e_{U}\hat{\xi }\right]dx$$

(44) $$\hat{D^{\prime }}=\int_{\Omega }\delta_{\varepsilon }^{2}\circ \phi \left[\hat{\xi }G(\phi ,I)-{\hat{\xi }}^{2}U^{2}+\lambda \hat{\xi }\kappa (\delta_{\varepsilon }^{2}\circ \phi \cdot \hat{\xi })\right]dx-\int_{\Omega }\delta_{\varepsilon }^{2}\circ \hat{\psi }\left[{\hat{\psi }}^{2}-e_{U}\hat{\xi }{\left(\alpha U\right)}^{2}\right]dx.$$

After adding Eq-43 to Eq-44 and combining the $\delta_{\varepsilon }^{2}\circ \hat{\psi }$ terms, the portion containing error *e_U_* can be conveniently factored:

(45) $$F^{\prime }+\hat{D^{\prime }}=\int_{\Omega }\delta_{\varepsilon }^{2}\circ \phi \left[\hat{\xi }G(\phi ,I)-\hat{\xi^{2}}U^{2}+\lambda \hat{\xi }\kappa (\delta_{\varepsilon }^{2}\circ \phi \cdot \hat{\xi })\right]dx-\int_{\Omega }\delta_{\varepsilon }^{2}\circ \hat{\psi }\left[e_{U}^{2}\alpha^{4}U^{4}-2{\left(\alpha U\right)}^{2}e_{U}\hat{\xi }+\hat{\xi^{2}}\right]dx.$$

(46) $$=\int_{\Omega }\delta_{\varepsilon }^{2}\circ \phi \left[\hat{\xi }G(\phi ,I)-\hat{\xi^{2}}U^{2}+\lambda \hat{\xi }\kappa (\delta_{\varepsilon }^{2}\circ \phi \cdot \hat{\xi })\right]dx-\int_{\Omega }\delta_{\varepsilon }^{2}\circ \hat{\psi }{\left[e_{U}{\left(\alpha U\right)}^{2}-\hat{\xi }\right]}^{2}dx.$$

When *U* and λ satisfy Theorem III.1, it follows that

(47) $$V^{\prime }=F^{\prime }+\hat{D^{\prime }}\leq -\int_{\Omega }\delta_{\varepsilon }^{2}\circ \phi \cdot \hat{\xi^{2}}+\delta_{\varepsilon }^{2}\circ \hat{\psi }{\left[{\left(\alpha U\right)}^{2}e_{U}-\hat{\xi }\right]}^{2}dx\leq 0.$$

Thus, *V*′(*t*) is negative semidefinite and *V*(*t*) bounded.

### D. Synthetic Image Example

**Figure 8:**
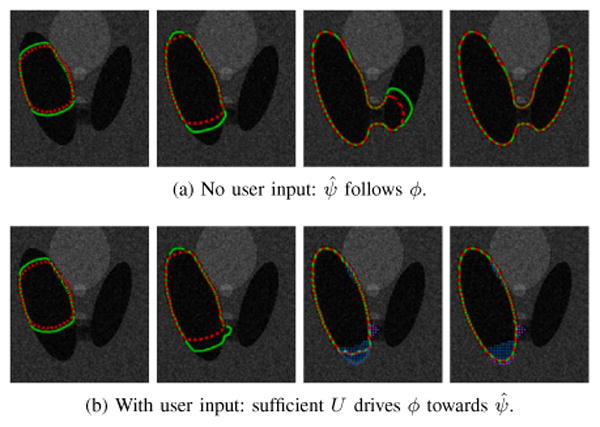
A synthetic image example. The human user seeks to segment only the left ellipsoid region; the open-loop system tends to creep through and segment the union of the two ellipsoids. |*ϕ*| ≤ *ε* and |*ψ̂*| ≤ *ε* are denoted by solid-green and dashed-red contours, respectively. Dotted regions in (b) indicate |*U*| > 0.

To demonstrate the coupled dynamics, this section considers a simple segmentation scenario. Fig-8 illustrates closed-loop system behavior on the synthetic image used previously in Section II-B. In the absence of user input, *ϕ*(**x**, *t*) behaves like the open-loop system; Fig-8a shows the *ψ̂* estimate following *ϕ* until they both reach steady state. With user input, the estimated ideal contour mediates between user input and the open-loop segmentation dynamics. In Fig-8b, the user starts to apply input upon noticing the *ϕ* = 0 boundary creeping through the bridge between the two ellipsoidal regions. Input stops and the system reaches steady-state after the user is satisfied with the displayed segmentation. Comparing Fig-8a and Fig-8b, we see that regardless of user input, the closed-loop sytem aligns the zero level-sets of *ϕ* and *ψ̂* at steady-state; the presence of user input in Fig-8b shifts the steady-state of *ϕ* and *ψ̂*. In both cases the *α* for Eq-39 is set to 1/*U*_M_.

## V. Application to MRI and CT Images

In this section, the feedback-augmented level-set methods are applied to two specific problems involving interactive medical image segmentation of X-ray Computed Tomography (CT) and Magnetic Resonance Imaging (MRI) volumes. In Section V-A, a fractured piece of the femur is segmented in a CT volume. Next, the technique is applied to extract a patellar tendon in an MRI volume in Section V-B. For both applications, we first review the clinical problem. Next, an open-loop system appropriate for the image type is chosen. Finally, the closed-loop *ϕ* system is summarized, followed by a discussion of the segmentation results.

### A. CT Segmentation with Mean-Alignment

**Figure 10:**
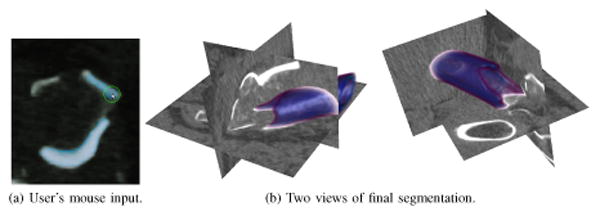
Segmentation of shattered hip bone fragments in a CT scan. The image volume is 156 × 162 × 229 voxels with a 0.7mm grid spacing.

The realignment of bone fragments after a fracture, also referred to as *fracture reduction*, is a crucial task during the operative treatment of complex bone fractures. Anatomically incorrect fracture reduction can result in severe post-traumatic complications. In order to avoid such problems and obtain an optimal fit between all relevant fracture fragments, the surgeon traditionally exposes the fractured bone by cutting the soft tissue envelope to access the fragments directly. Subsequent realignment of the recovered fracture fragments requires a trial and error approach, which prolongs surgery and increases the risk of complications for the patient. Therefore, there is a clear need for the development of less invasive techniques to reconstruct complex fractures. Segmentation of the image data to localize the fragments (as in Fig-10) is a key step first step toward computing and planning the optimal way of realigning the fractured bone.

Bone tissue generally appears very bright in CT imagery; therefore, the segmentation of bone in CT is modeled with the mean-alignment system Eq-9. Using the control from Eq-13 leads to the closed-loop system

(48) $$\phi_{t}=\delta_{\varepsilon }\circ \phi \left[-{\left(I-\mu_{\text{in}}\right)}^{2}+{\left(I-\mu_{\text{out}}\right)}^{2}-\hat{\xi }U^{2}+\lambda (t)\kappa (\delta_{\varepsilon }^{2}\circ \phi \cdot \hat{\xi })\right].$$

Note that a healthy bone can often be segmented in its entirety using the open-loop system alone, since the zero level-set of *ϕ* is naturally drawn to boundaries of bright objects. Interactive control becomes vital, however, when segmenting bone subject to disease or injury; accurate segmentation is not possible without feedback.

**Figure 11:**
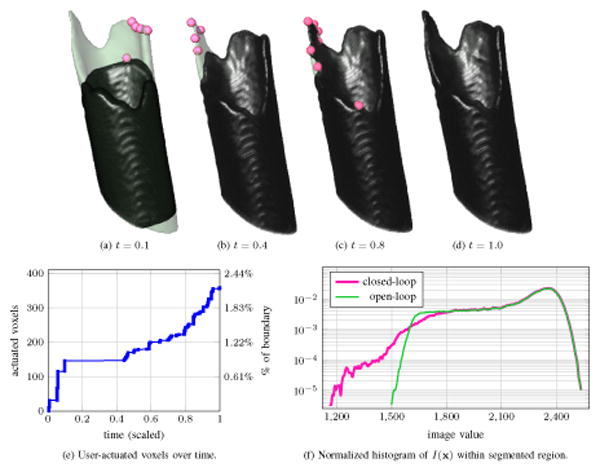
In (a)-(d), regions of user input are shown as markers on the progressing segmentation (dark) overlayed on user's reference boundary (light). The segmentation in Fig-11a is the steady state of the open-loop system. In Fig-11d, the segmentation agrees with the desired reference boundary due to the closed-loop system's incorporation of user input.

Fig-11 illustrates several aspects of the interactive system applied to the segmentation of a large bone fragment. Local maxima of *U*(**x**) are shown as markers along with the intermediate *ϕ* = 0 boundary in Fig-11 (a)-(d); the green semi-opaque surface represents the user's reference *ψ* = 0 level-set. Regions of bright CT image values are quickly segmented by the open-loop system; the user generates some input along what appears to be an edge of the fractured bone where *I*(**x**) is darker (Fig-11a). After the system has segmented this first edge and is nearly at steady-state, the user finds another edge along which to apply input (Fig-11b). A further refinement is made in Fig-11c that leads to the final steady-state segmentation of Fig-11d. The number of voxels actuated by the user's mouse strokes are plotted versus (scaled) time in Fig-11e. In a fully manual segmentation, each of the 16404 boundary voxels in Fig-11d would need to be marked by the user; the second *y*-axis on the right indicates the actuated voxels as a percentage of the fully manual effort. It is difficult for a person to accurately decide whether or not a fracture edge in a distant part of the volume is part of the same fragment Fig-11e indicates that a substantial portion of time is spent with *ϕ* near a steady-state while the user scrolls through slices to decide where fracture edges are located and whether these edges are part of the same fragment or another one in close proximity In Fig-10a two light regions are determined to be part of the fragment being segmented while a third (in the upper left) is a separate bone fragment.

Normalized histograms of the image intensity distribution inside of the segmentation boundary at steady-state are shown in Fig-11f Without feedback the segmentation encloses a region with a highly peaked *I*(**x**) histogram In contrast the closed-loop system reaches steady-state with a heavier-tailed intensity histogram The distribution shift is due to the user applying input to correctly label bone near and along the jagged fragment edges These regions are precisely where we care most about accurate segmentation since the fragment's edges are to be matched with those of other fragments during the fracture reduction task.

### B. MRI Segmentation with Localized Statistics

Surgical repair of a torn anterior cruciate ligament (ACL) requires choosing a location from which to harvest a graft of sufficient length and thickness. The most common choice today is the patellar tendon (PaT). While the width and thickness of a PaT are quite predictable based on patient height and weight, the tendon length varies widely. This variability in shape continues to complicate surgery due to mismatch between the graft and drilled tunnel, especially in “anatomic reconstruction” [51]–[53] where the replacement ACL is to be oriented exactly as before the injury^2^. Quantifying the variability of PaT shape and comparing it to other graft choices (namely the hamstring and quadriceps tendons) requires accurate segmentation in MRI volumes.

**Figure 12:**
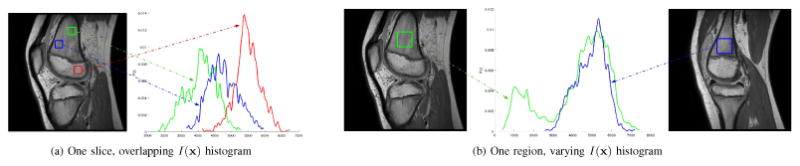
Tissues within one MRI slice have overlapping intensity histograms while a single tissue across slices has a varying histogram. Separation of regions must consider the spatially-varying image statistics.

Soft tissue including tendons and ligaments is readily visible in MRI, unlike CT where only mineral-dense bone gives a strong response. However, images obtained by MRI have a complicated mapping between tissue type and observed intensity; segmenting soft tissue in MRI is generally more difficult than bone in CT. The distribution of intensity values in MRI arising from a particular anatomic structure will vary significantly between slices (Fig-12b), and will also overlap the distributions of other structures (Fig-12a). An effective approach for MRI segmentation is to separate regions based on spatially-varying statistics of *I*(**x**). To do so, open-loop dynamics are chosen to use the localizing active contours of [54] that define intensity means *μ_in_*(**x**) and *μ_out_*(**x**) locally as integrals over a Euclidean ball of radius *r*. With feedback the system becomes

(49) $$G(\phi ,I)=\int_{B_{r}(x)}\delta_{\varepsilon }\circ \phi (y)\left[{\left(I(y)-\mu_{\text{in}}(x)\right)}^{2}-{\left(I(y)-\mu_{\text{out}}(x)\right)}^{2}\right]dy,$$

(50) $$\phi_{t}=\delta_{\varepsilon }\circ \phi \left[G(\phi ,I)-\hat{\xi }U^{2}+\lambda (t)\kappa (\delta_{\varepsilon }^{2}\circ \phi \cdot \hat{\xi })\right].$$

**Figure 13:**
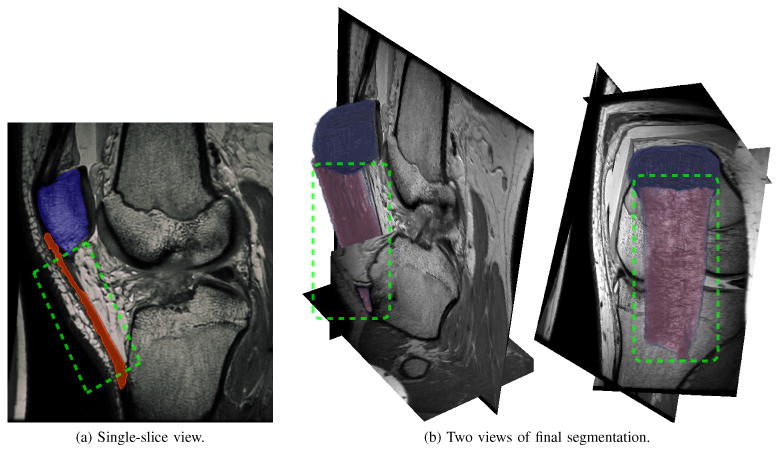
A segmentation of the patella and patellar tendon in MRI, part of a study on graft selection for anterior cruciate ligament (ACL) repair. The image volume is 512 × 512 × 224 voxels with a 0.4mm grid spacing.

Despite the advantages of the underlying open-loop system segmenting a PaT remains challenging for two reasons First, the tendon is very thin relative to its height and width making a satisfactory choice of *r* in Eq-49 difficult Second, *I*(**x**) at the insertion points of the tendon has the same distribution locally as adjacent connective tissue The human user however employs their anatomic knowledge to enable successful segmentation via the closed-loop system Fig-13 shows the final result; the tendon has been segmented between its attachment on the inferior pole of the patella to the end of its insertion on the tibial tubercle For context the patella bone is also segmented and displayed.

**Figure 14:**
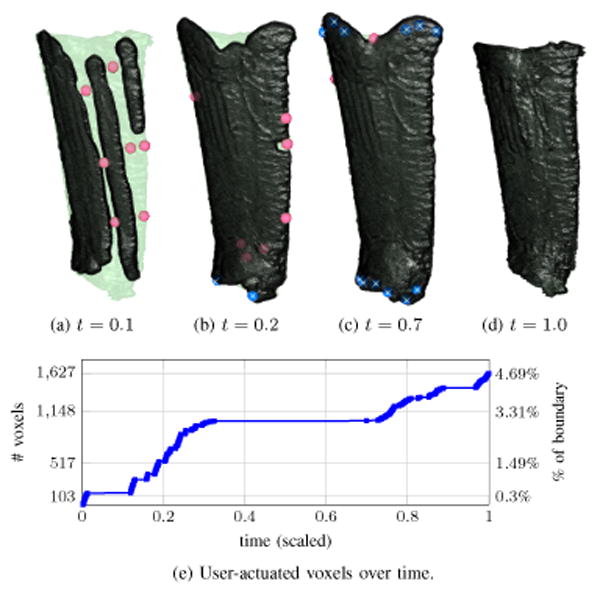
Regions of significant user input shown together with the changing segmentation. Locations where *U* > 0 and *U* < 0 correspond to red and blue markers, respectively. The open-loop system's tendency towards “bleed-through” near the insertion points is handled in the closed-loop system by incorporating user input with negative *U*.

Incremental progress during interactive segmentation of the tendon is shown in Fig-14 (a)-(d) Red and blue markers denote positive and negative extrema of *U*, respectively while the green semi-opaque surface represents a reference segmentation known to the human expert As the *ϕ* = 0 boundary evolves after initialization a small amount of input yields the segmentation in Fig-14b With the bulk of the tendon outlined the user applies input to fill gaps in the vertical edges and remove the over-segmented regions around the insertion points at the patella and tibia bones (Fig-14c) Unlike the fracture scenario in Fig-11 the open-loop system applied to segment this tendon leads to massive “bleed-through” of the segmentation because the image distribution around the tendon insertion points is identical to that of the tendon itself Fig-14d shows the steady-state reached by the closed-loop system; user input stabilizes the segmentation at the desired reference boundary and prevents bleed-through past the insertion points on the patella and tibia. In Fig-14e, the number of voxels actuated by the user's mouse strokes is 4.7% of what would be needed to trace all of the tendon's boundary voxels manually. Comparing Fig-11e and Fig-14e, the latter has more piecewise constant regions because the human user spends substantial time looking for anatomic markers and adjusting displayed image contrast to decide where the tendon begins and ends.

## VI. Comparison to Related Methods

### A. Overview

In many implementations of level-set segmentation (e.g. [11], [17], [20]), the smoothing factor λ is a parameter that is set by a user. However, understanding such a parameter requires users to have more mathematics background than is typical for the medical community. Here, we set λ automatically to achieve desired behavior. The PDE control formulation here has a constant computational cost with respect to amount of user input and no abrupt changes to the segmentation, unlike in [21]–[23]. Under the proposed controller, sufficient input *U*(**x**) from the user guarantees agreement with the reference state; relaxed constraints in [22] dictate that it may be impossible for the segmentation to respect the user's inputs. Rushed use of the mouse by a human is not possible in [21], [23] because constraints are exactly enforced. In contrast, the input processing used in the current work provides leeway for the user: a small |*U*| will not dominate the open-loop dynamics. If needed, a large accumulation of |*U*| is achieved by “scribbling” repeatedly in a region.

It is emphasized that the closed-loop control formulation in this paper does not seek to replace existing curve evolution algorithms but rather to augment them. The control-theoretic approach enables a user to reach the desired segmentation at steady-state; running the level set evolution for a longer time will not cause the boundary to “bleed-through” or contract.

### B. Quantitative Comparison with GrabCut

**Figure 15:**
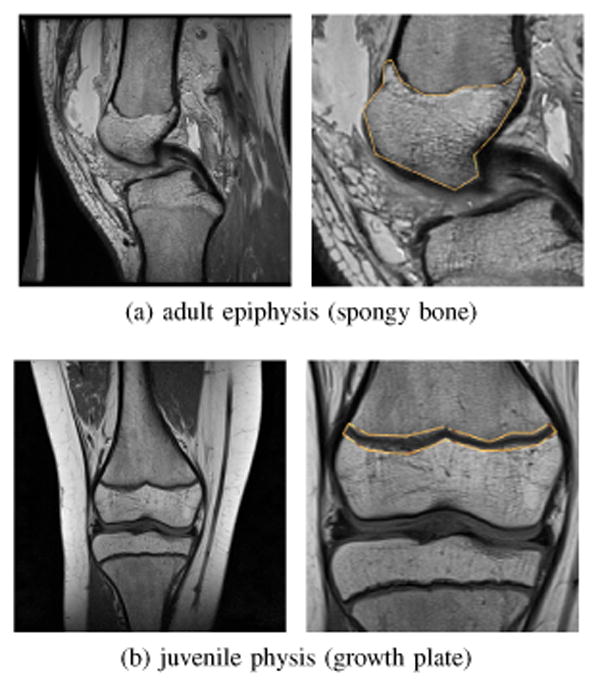
Two test images are used in a quantitative comparison of GrabCut and the proposed algorithm. Regions of interest are outlined above; several interactive segmentations of each region are performed.

Two orthopedic images are used as test data to quantitatively compare the method presented in this paper with the popular GrabCut algorithm [19]. The user's goal is to segment the epiphysis and physis of the femur in Fig-15a and Fig-15b, respectively. User input via mouse click-and-drag is implemented and measured identically for both algorithms. The GrabCut implementation used here is available as part of the OpenCV library [55]. A location through which the cursor was dragged is defined as an “actuated voxel;” the extents around the cursor that mark seed regions in GrabCut are not counted towards this total. Locations in the image whose assigned label changes between background and foreground are tracked over time and are referred to as “reclassified voxels.” In both implementations, total segmentation time is primarily a function of how long it takes the user to evaluate the current state and apply more corrective mouse strokes. The total number of actuated voxels needed to complete the segmentation is a robust indicator of user effort.

**Figure 16:**
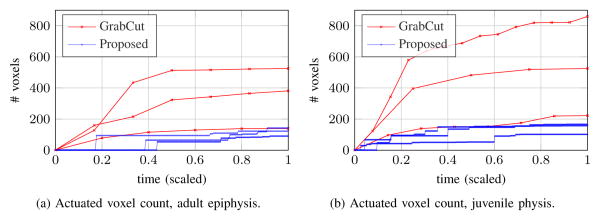
Comparison of actuated voxels over time, after initialization. The proposed algorithm has both a lower mean actuated count and tighter clustering across repeated segmentations.

Actuated voxels after initialization are plotted in Fig-16. At termination, all of the segmentations have greater than 98% overlap with a manually created reference. For the adult epiphysis image of Fig-15a, the average final actuated voxel counts are 348 (GrabCut) and 118 (proposed). For the juvenile physis segmentation, the averages are 536 (GrabCut) and 141 (proposed). Segmenting the physis is more difficult with GrabCut due to the elongated shape, the nearly identically-looking fluid around the bone and the bimodal appearance of cortical bone above and spongy bone below the physis. A GrabCut iteration can change the segmentation dramatically; when this change is erroneous, significant corrective effort becomes required. In Fig-16b, we see this manifested by the large increases in actuated voxels during the first few rounds of GrabCut user input. In contrast, the proposed algorithm provides rapid continuous visual feedback for the user; small corrections are made before a large error can develop.

**Figure 17:**
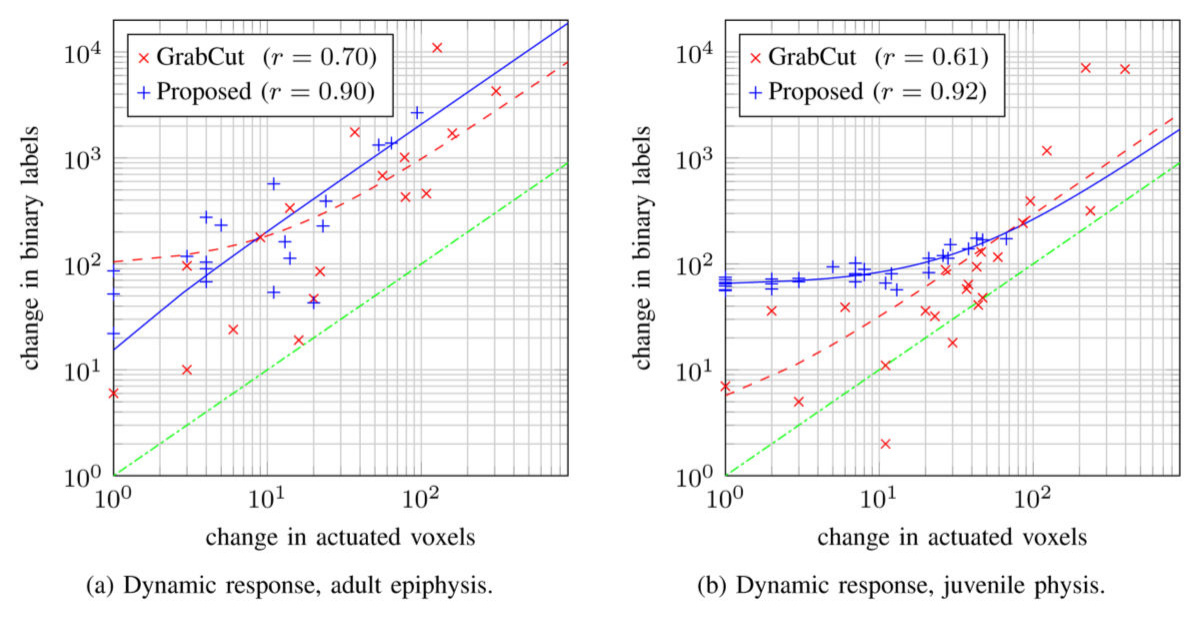
Comparison of dynamic response to user input; data points and linear fit lines are shown. Points below the dashed green line indicate wasted user effort since more additional voxels were actuated than reclassified.

Predictability of how the segmentation changes in response to mouse strokes is a criterion for practical ease of use. Two scatterplots quantify the predictability in Fig-17; dynamic response is characterized in terms of the number of reclassified voxels (*Y* -axis) and the number of newly actuated voxels (*X*-axis). Each mark corresponds to one iteration when new user input was applied. Linear regression lines are overlaid on the data. The two algorithms have a similar dynamic response in the epiphysis segmentation, shown in Fig-17a; correlation coefficients are 0.70 (GrabCut) and 0.90 (proposed algorithm). Two issues become apparent in Fig-17b for the juvenile physis scenario. First, the distribution of GrabCut data points is quite broad; correlation coefficients are 0.61 and 0.92 for GrabCut and the proposed algorithm, respectively. Second, many of the GrabCut data points are below the dashed green line, indicating a waste of user effort since there are more voxels actuated than reclassified. The dynamic response of GrabCut makes it hard for a user to predict how much change new mouse strokes will cause.

## VII. Conclusion

This paper has presented a modeling approach that enables control-theoretic analysis and design for interactive medical image segmentation. Results shown for a synthetic image (Section IV-D) and real medical volumes (Section V) agree with theoretical expectations of system performance. Section V illustrated two qualitatively different situations: (1) gradual expansion of the boundary to bound the entire femur fragment and (2) prevention of “bleed-through” or over-segmentation with the patellar tendon. In both situations, the user is able to drive the segmentation to a desired steady state and to do so with much less effort in terms of actuated voxels than manual segmentation. In summary, the PDE control formulation enables us to guarantee a user's ability to reach a reference segmentation state while also absolving them of the need to understand mathematical details or use precise mouse movement.

Successful use of the closed-loop algorithm by medical students motivates several future extensions. If a single image contains several objects of interest, they would need to be extracted sequentially in the current framework. Such a sequential de-coupled approach does not address natural constraints of the geometry and involves re-editing common boundaries. A coupled formulation using an open-loop system of PDEs such as in [20] together with a vector of control inputs would prevent region overlap and reduce the user's effort when segmenting multiple regions. Informative visualization is vital for efficient interaction, since performance of any interactive segmentation method is limited by how quickly and accurately a user can infer the segmentation state [56]–[58]. An interesting extension to the theory would consider the feedback between visualization and the creation of user input; for example, it may be desirable to confine movement of the boundary to regions that are observable from the user's viewpoint in 3D.

## Figures and Tables

**Figure 9:**
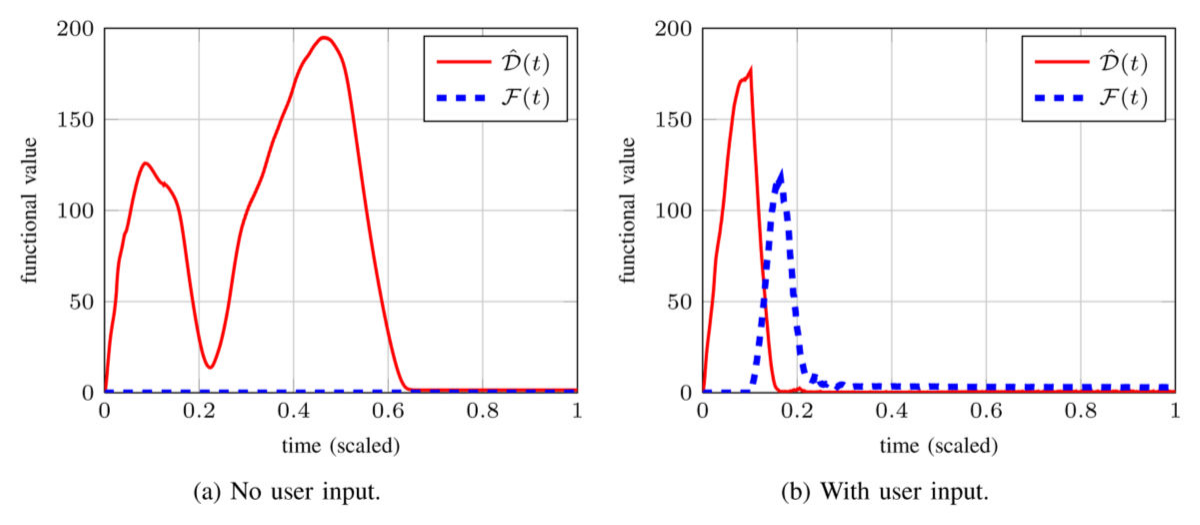
Shown above are the values of 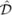 and 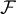 corresponding to the synthetic image example of Fig-8. *Left*: *ϕ* is driven only by the image-dependent term in the absence of user input. *Right*: 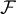
(*t*) rapidly grows when the user applies input. 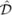
(*t*) rapidly shrinks as *ϕ* is drawn towards *ψ̂*. At steady-state, 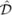
(*t*) < 0.5 and 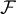
(*t*) < 3.

## Footnotes

1 The dimension is *n* = 3 or *n* = 2, for volumetric images and planar images, respectively.

2 as opposed to the more common approach of attaching a shorter graft to an arbitrary reachable point on the bone surface.
